# A Smartphone-Based Detection System for Tomato Leaf Disease Using EfficientNetV2B2 and Its Explainability with Artificial Intelligence (AI)

**DOI:** 10.3390/s23218685

**Published:** 2023-10-24

**Authors:** Anjan Debnath, Md. Mahedi Hasan, M. Raihan, Nadim Samrat, Mashael M. Alsulami, Mehedi Masud, Anupam Kumar Bairagi

**Affiliations:** 1 Department of Computer Science and Engineering, North Western University, Khulna 9100, Bangladesh; anjandebnath.cse@gmail.com (A.D.); mdmahedihasan76@gmail.com (M.M.H.); nadimsamrat.nwu@gmail.com (N.S.); 2 Department of Information Technology, College of Computers and Information Technology, Taif University, Taif 21944, Saudi Arabia; mashael.s@tu.edu.sa; 3 Department of Computer Science, College of Computers and Information Technology, Taif University, Taif 21944, Saudi Arabia; mmasud@tu.edu.sa; 4 Computer Science and Engineering Discipline, Khulna University, Khulna 9208, Bangladesh

**Keywords:** tomato leaf, deep learning, transfer learning, EfficientNetV2B2, explainable AI, LIME, Grad-CAM, ablation study, smartphone, web application

## Abstract

The occurrence of tomato diseases has substantially reduced agricultural output and financial losses. The timely detection of diseases is crucial to effectively manage and mitigate the impact of episodes. Early illness detection can improve output, reduce chemical use, and boost a nation’s economy. A complete system for plant disease detection using EfficientNetV2B2 and deep learning (DL) is presented in this paper. This research aims to develop a precise and effective automated system for identifying several illnesses that impact tomato plants. This will be achieved by analyzing tomato leaf photos. A dataset of high-resolution photographs of healthy and diseased tomato leaves was created to achieve this goal. The EfficientNetV2B2 model is the foundation of the deep learning system and excels at picture categorization. Transfer learning (TF) trains the model on a tomato leaf disease dataset using EfficientNetV2B2’s pre-existing weights and a 256-layer dense layer. Tomato leaf diseases can be identified using the EfficientNetV2B2 model and a dense layer of 256 nodes. An ideal loss function and algorithm train and tune the model. Next, the concept is deployed in smartphones and online apps. The user can accurately diagnose tomato leaf diseases with this application. Utilizing an automated system facilitates the rapid identification of diseases, assisting in making informed decisions on disease management and promoting sustainable tomato cultivation practices. The 5-fold cross-validation method achieved 99.02% average weighted training accuracy, 99.22% average weighted validation accuracy, and 98.96% average weighted test accuracy. The split method achieved 99.93% training accuracy and 100% validation accuracy. Using the DL approach, tomato leaf disease identification achieves nearly 100% accuracy on a test dataset.

## 1. Introduction

Crops, the foundation of human nourishment, are essential to feeding the world’s population [1]. There are many different types of crops, but none stand out as much as tomatoes [2]. Tomatoes account for around 15% of all vegetables consumed globally, with an astounding 20 kg yearly per capita consumption rate [3]. The output of fresh tomatoes exceeds 170 million tons annually, making it the most plentiful vegetable crop worldwide [4]. China, Egypt, Turkey, the United States, and India are the top tomato producers, demonstrating its widespread use and economic importance [5].

Tomato cultivation is widely practiced, although it is not without its difficulties [6]. The sneaky existence of tomato leaf diseases is the biggest threat to the worldwide tomato business [7]. The Food and Agriculture Organization of the United Nations reports that these diseases substantially negatively impact tomato production globally, with an annual loss rate of up to 10% [8]. The tendency of these diseases to start in the leaves before ferociously spreading across the entire plant makes them even more worrisome [9].

Historically, diagnosing and treating these disorders has been a time-consuming and expensive process that frequently relied on manual examinations by qualified professionals [10]. But with the advent of the digital era, agriculture has undergone a fundamental change [11]. Automated AI image-based solutions have become an essential weapon in the battle against illnesses affecting tomato leaves [12]. The advent of cutting-edge software and technologies has ushered in an era when pictures are acknowledged and used as a reliable method of disease diagnosis [13]. This innovation uses image processing, an intelligent method that boosts recognition precision, lowers expenses, and improves picture recognition efficiency [14].

Computer vision technology is one of the most effective and ecologically responsible ways to identify plant diseases [15]. This technique provides a low-cost, non-destructive method of spotting agricultural problems with no negative effects on the environment [16]. Particularly obvious symptoms of underlying plant diseases are the scars and abnormalities that appear on leaves [17]. Healthy leaves have a consistent color and texture, but sick leaves show differences and frequently have recognizable patterns of illness spots [18]. To improve illness diagnosis, researchers have explored a variety of imaging methods and tailored illumination conditions in labs [19].

Although they can be useful in some cases, conventional diagnostic techniques are burdened by their high cost and proneness to human mistakes [20]. On the other hand, the quick development of computer technology has given rise to creative solutions. In the identification of agricultural diseases, computer vision, machine learning (ML), and DL have found their place. These tools make it possible to separate RGB photos of crop diseases depending on their color, texture, or form characteristics [21]. Even in complicated natural contexts where several illnesses may display identical symptoms, our advanced technique significantly improves the accuracy of disease diagnosis [22]. The study has significant ramifications for sustainable agriculture, global food security, and technology’s crucial place in contemporary farming methods.

ML and DL models have an influence beyond tomatoes [23]. These tremendous instruments have the potential to revolutionize agriculture in general [24]. We can equip farmers with the tools they need to protect their crops, improve food security, and strengthen the world’s agricultural economy by modifying and training these models to detect particular crops’ distinctive traits and illnesses [25]. This revolutionary use of technology promises improved precision and a more sustainable and successful future for agriculture globally [26]. As we progress further into the digital era, the interdependence of agriculture and technology will become increasingly crucial to ensure the success of our crops and plenty of our meals [27]. Nowadays, artificial intelligence (AI)-based expert systems (smartphone applications, web applications) are more useful for this detection. So, if it is possible to implement the detection system in a smartphone application, this would be more powerful and easy for everyone to use. So we thought about the concept and worked on it to implement the detection process on smartphones and web applications. Anyone can take pictures of tomato leaves, and a smartphone application or web application can be used to obtain promising results. The major contributions of this work are as follows:

- We optimize a very effective DL model, EfficientNetV2B2, for tomato leaf disease detection.
- The proposed model is evaluated using different matrices such as loss curve, ROC curve, confusion matrix, precision, recall, F1-score, and accuracy with datasets [28,29]. The model is also justified by comparing it with state-of-the-art deep learning models and customized models [30,31,32,33,34,35,36].
- A smart application system has been built to detect and classify tomato leaf diseases, adapting to both smartphone and web-based interfaces. The application provides the results in both English and Bangla.
- The explainable AI frameworks such as LIME and Grad-CAM are also used to analyze the model.

The subsequent sections of this work are structured in the following manner. Section 2 covers the Literature Review, whereas Section 3 presents the Methodology of this investigation. Section 4 provides a comprehensive account of the Experimental Outcomes and Discussions, while Section 5 is a summary of our findings and conclusions.

## 2. Literature Review

Agarwal et al. [30] implemented a convolutional neural network (CNN) using the dataset from [29]. This dataset is vast and includes many types of crops. However, in this particular experiment, tomato leaves were only utilized. A cohort consisting of 10 individuals was employed, and a dataset including 10,000 photographs was utilized for training purposes. In order to ensure the accuracy and reliability of the results, a validation approach was employed, wherein 700 instances were allocated for each class, while 50 instances were assigned for each kind for testing purposes. The dimensions of the image were 256 × 256. The model was executed for a total of 1000 epochs. The researchers attained a mean test accuracy of 91.20%. The present study involved the development of a convolutional neural network (CNN) model for the purpose of detecting diseases in tomato crops. The architecture has three convolution and maximum pooling layers, each characterized by a distinct number of filters. One notable feature of the proposed model is its minimal storage requirement of approximately 1.5 MB, in contrast to the significantly larger storage demand of around 100 MB for pre-trained models. Subsequent investigations will endeavor to refine the model by using a more extensive dataset comprising a greater quantity of photographs encompassing diverse cropping techniques.

Similarly, Ahmad et al. [37] tried laboratory-based tomato leaves collected from a repository. They used only four classes of tomato leaves, splitting the dataset into training (70%), validation (20%), and testing (10%). They also used different deep learning models. Among them, using feature extraction, Inception V3 achieved the best accuracy of 93.40%, and using parameter tuning, Inception V3 achieved the best accuracy of 99.60%. They found that feature extraction produces less accurate outcomes than parameter adjustment. The future logical progression of their work will be to improve these models’ performance on actual field data.

Zhao et al. [31] used the plant village dataset [29] and selected only tomato leaves of 10 classes. Image size used 224 × 224. The SE-ResNet50 model achieved the best average of 96.81 accuracy in the experiment. A multi-scale feature-extraction model was developed for the purpose of identifying tomato leaf diseases. Subsequent research endeavors will encompass the timely automated detection of tomato and other agricultural ailments through the utilization of these trained models. The researchers will also employ the proposed approach to automate the identification of tomato leaf diseases in an authentic agricultural environment, employing a greenhouse inspection robot that was created independently by the team.

Zhou et al. [38] used tomato leaf disease datasets comprising 13,185 images with nine classes. The image size used was 196 × 196 pixels. The dataset was split into training (60%), validation (20%), and testing (20%). Deep CNN, ResNet50, DenseNet121, and RRDN were used and achieved the best accuracy on the RRDN model at 95%. In this study, residual dense networks were recommended for tomato leaf disease detection. They changed the model architecture to create a classification model with higher accuracy than cutting-edge methods. They hope to use these findings to improve agricultural intelligence.

Trivedi et al. [39] used tomato leaf disease datasets, where nine types were classified as infected and one class was resistant. Images were normalized by setting a resolution of 256 × 256 pixels. Then, the images were changed to grey. A convolutional neural network was tried with different epochs and different learning rates. Finally, they achieved the best accuracy at 98.58%, and the detection rate of that model was 98.49%. The study examined a deep neural network model that accurately detected and classified tomato leaf diseases. The crop leaf lacked nutrients, thus the model was expanded to incorporate other abiotic illnesses. The researchers wanted to maximize data collection and learn about various plant diseases. New technologies will improve precision in the future. Wu et al. [40] collected a dataset from Plant Village [29] and used only tomato leaves for this experiment. They tried five different classes. For GoogLeNet, AlexNet, and ResNet, they used an image size of 224 × 224 pixels, and for VGG, they used an image size of 299 × 299 pixels of RGB color space. A total of 1500 images were used for this experiment. This experiment used AlexNet, GoogLeNet, ResNet, and VGG16, and among them, GoogLeNet achieved the best accuracy of 94.33%. They also tried DCGAN, BEGAN, and DCGAN + BEGAN, and among them, DCGAN achieved the best 94.33% accuracy, but the accuracy on the test was 67%. This experiment tried to find different accuracies using different learning rates. In this study, the authors showed that DCGAN can produce data that closely resemble genuine photos, hence increasing the size of the dataset for training big neural networks, enhancing the generalizability of recognition models, and increasing the variety of data. To recognize tomato leaf disease, they intend to develop a better data-augmentation approach in the future. This will increase the recognition’s robustness and accuracy.

Chen et al. [19] collected a dataset of tomato leaves from the Hunan Vegetable Institute. Images were taken in natural light, and the image size was 4460 × 3740. They collected a total of 8616 images of five kinds of diseases. They tried it with the B-ARNet model architecture, and using a 224 × 224 image size, the model achieved an accuracy of 88.43%. Then, they compared it with ARNet, ResNet50, and AlexNet. Among all of them, their B-ARNet achieved the best accuracy at 88.43%. This article suggests a strategy for identifying tomato leaf disease based on the ABCK-BWTR and B-ARNet models. There are few studies on the identification of multiple diseases on the same blade, despite the B-ARNet recognition model’s suggestion that they can improve the recognition effect of tomato diseases, particularly similar diseases under complicated backgrounds. To increase the model’s capacity for generalization, the image data of tomato leaf disease should be progressively expanded in the future.

All of these studies show what happens to infected tomato leaves when different models are used. It accurately predicted tomato disease leaves in certain studies, even when learning rates and epochs were altered. A summary of the literature review is implemented in Table 1.

## 3. Methodology

**Figure 1:**
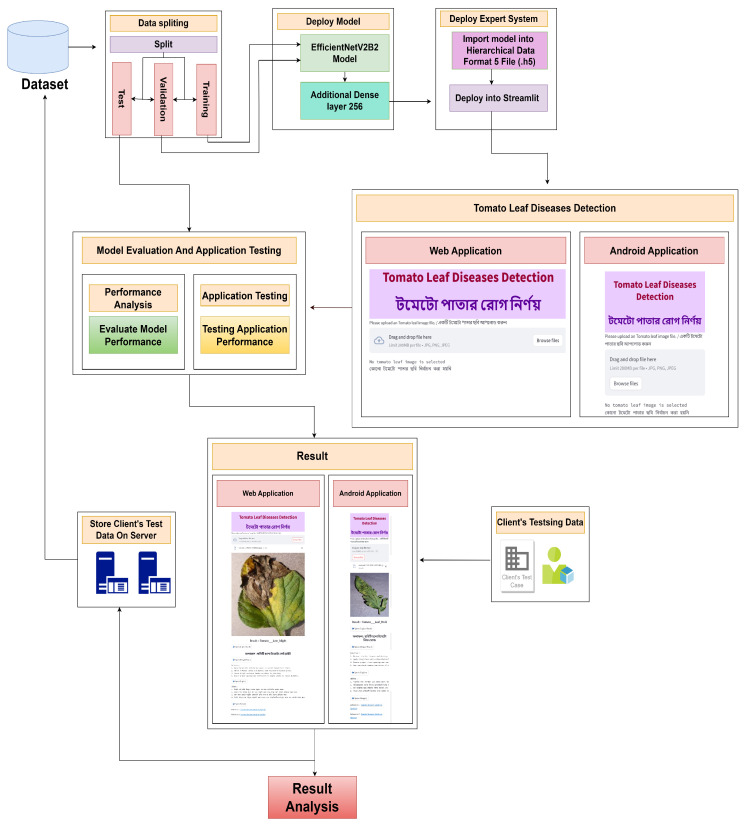
The workflow for tomato leaf disease detection with the suggested user application.

Figure 1 shows that we first collected the dataset and split it into training, testing, and validation sets. Then, we deployed the model EfficientNetV2B2 with an additional dense layer 256 and finally built an expert system using the model. We created both a web application and a smartphone application that will take tomato leaves as input and produce results. In the background, we collect these images and train our model with those images because they are now are training dataset. This process will continue as a loop to keep our detection system updated.

### 3.1. Dataset

**Figure 2:**
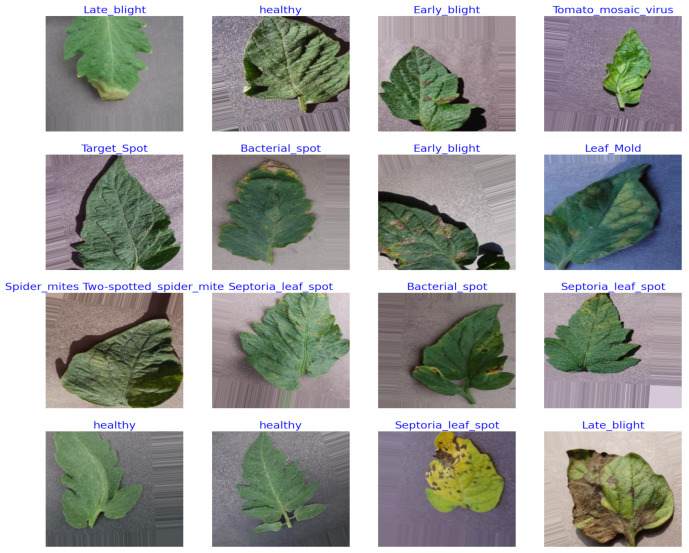
Randomly selected disease-affected tomato leaves from the dataset.

Every stage in the experiment requires datasets. Ten thousand images of tomato leaves were obtained from Kaggle [28] for the following dataset. We also stored the dataset on https://zenodo.org/record/8311631 (accessed on 2 September 2023) for further use. There is also the smartphone application file (.apk), which we created in the smartphone application folder. The diseases and their corresponding number of samples are shown in Table 2. This is a ten-class dataset with one healthy leaf class. Each class contains 1000 samples, and hence, this is a balanced dataset. Figure 2 displays a few samples from this dataset that were picked randomly. The images were processed with a 256×256 resolution.

### 3.2. Data Split

The repository for “Tomato leaf diseases detection” [28] is where samples of tomato leaf disease were found. An RGB color space and the JPG file format were used to store images, which have 256×256 resolution. We divided the dataset into three sets: 8000 images for training (800 images in every class), 1000 images for the validation set (100 images from each class), and 1000 images for the test set (100 images from every class).

### 3.3. InceptionV3 Architecture

A crucial convolutional neural network (CNN) architecture for image identification is InceptionV3 [41], which was created by Google Research in 2015. It can collect features at various scales and achieve exceptional accuracy because of the creative “Inception modules” that include parallel convolutional filters of various sizes. Deep learning in computer vision has significantly advanced due to the widespread adoption of InceptionV3 in image-categorization applications.

### 3.4. Convolutional Neural Network (CNN) Architecture

An essential deep learning architecture designed specifically for processing visual input is the convolutional neural network (CNN) [42]. They effectively perform tasks including picture classification, object identification, and facial recognition because of the novel usage of convolutional layers, which enables autonomous feature extraction. With its capacity to learn hierarchical features directly from raw pixel data, CNN has transformed the field of computer vision and is now the driving force behind applications in autonomous cars, medical imaging, and more.

### 3.5. EfficientNet Architecture

A deep convolutional neural network architecture family known as EfficientNet [43] has drawn attention to striking an outstanding balance between model accuracy and efficiency. EfficientNet, which was founded by Google Research in 2019, addresses the issue of scaling neural networks to improve performance while preserving the computing economy. The models in the EfficientNet series, which range from EfficientNet-B0 to -B7, each have a unique set of parameters and level of computational complexity. These models employ a compound scaling technique that balances the network’s depth, breadth, and resolution to achieve optimal performance in a range of computer vision applications. EfficientNet models have repeatedly performed the best in picture classification problems, showcasing their adaptability and effectiveness in a variety of real-world applications, including object identification, image recognition, and more.

### 3.6. Architectures of EfficientNetV2

**Figure 3:**
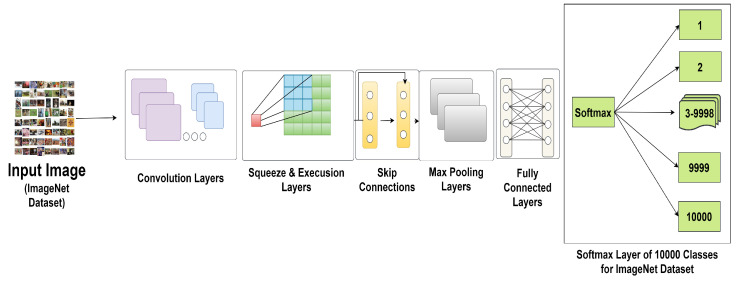
Model architecture for EfficientNetV2.

The Google Research-developed EfficientNet family of neural network topologies has evolved into EfficientNetV2 [23]. Compared to the original EfficientNet models, it represents a refinement and enhancement. V2-Small, V2-Medium, V2-Large, and V2-Extra Large are the sizes of the models in the EfficientNetV2 series. It also explains the versions of EfficientV2B0 to EfficientNetV2B3. Figure 3 shows the model architecture of the EfficientNetV2 model. By combining innovative architectural improvements, such as a new layer normalizing method termed “Ghost Batch Normalization” and an improved training pipeline, EfficientNetV2 improves on the success of its predecessor. With these modifications, training stability and performance are improved for a variety of tasks, including object identification, image classification, and more. The appealing combination of economy and accuracy that EfficientNetV2 models provide makes them appropriate for both resource-constrained and high-performance computing contexts.

Some of the blocks of the EfficientNet V2 architecture include

- **MBConv Block:** Mobile Inverted Bottleneck Convolution, the main component of EfficientNet, is represented by this. Squeeze-and-excitation procedures and depthwise separable convolution are also included.
- **Stem Block:** This is the first node in the network, and it is in charge of analyzing the input picture and extracting key information.
- **Block1, Block2, Block3, …:** These are the next blocks in the network, usually sorted in ascending order, with Block1 being nearer the input and higher-numbered blocks being further in the network.
- **Head Block:** The output layer and final predictions are handled by this network’s last building piece.

### 3.7. Deployed Model

**Figure 4:**
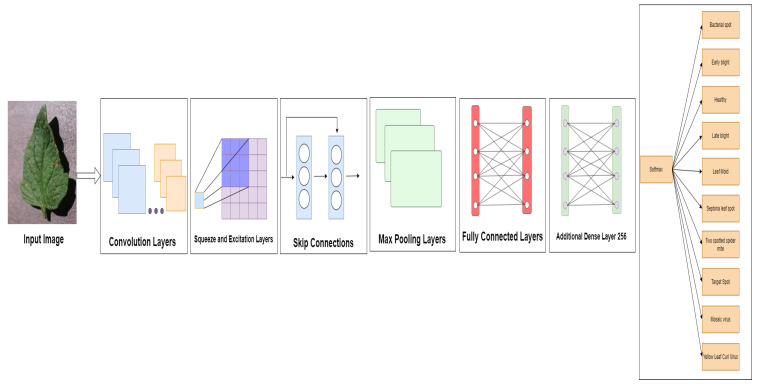
Model Architecture for EfficientNetV2B2 with Additional Dense Layer 256.

The pre-trained EfficientNetV2B2 [23] model, which was trained using the ImageNet dataset and 1000 category objects shown in Figure 3, was the method used for this experiment. As shown in Figure 4, to have the outputs match the classes in the dataset, we next reshaped the EfficientNetV2B2 model’s last layer with fully connected layers, one additional dense layer of 256, and 10 fully connected SoftMax layers. Table 3 shows the hyperparameters used in the EfficientNetV2B2 model.

- As the first step, the pre-trained model is shown.
- We used a fine-tuning approach and trained the model to classify the various tomato leaf diseases, including healthy leaves, by reshaping the final layer of the EfficientNetV2B2 model with fully connected layers and an additional dense layer of 256, then adding 10 fully connected SoftMax layers.

### 3.8. User Application Design

To create a user-friendly application, the top-performing EfficientNetV2B2 model from an experimental situation was deployed into a .H5 file. Here, the .H5 file extension, which stands for Hierarchical Data Format version 5, and the .H5 file are then integrated with the web application using the Python web framework Streamlit. Additionally, a user-friendly web application and Android app were created so that end users could quickly input the image to test the diseases of tomato leaves. The application architecture is also employed in both Bangla and English, making it easier for Bangladeshi farmers who require the program. It shows the class name of the diseases and suggests some solutions for them in both Bangla and English. After every result and explanation, we added some buttons to read the texts for the users in English and Bangla. This will be very helpful for many users who need to improve in reading English or Bangla but are good at listening. The solutions were collected from authentic sources, and reference links were added to both the web and the smartphone applications.

## 4. Experimental Outcomes and Discussions

Here, in this section, all the results of this experiment are shown, including the loss curve, accuracy curve, Confusion Matrix, and ROC curve, and the results are also compared with those of different approaches.

### 4.1. Cross-Validation Outcome of the EfficientNetV2B2 Model

A common method in ML for evaluating models is five-fold cross-validation. By offering several performance indicators across various data subsets, five-fold cross-validation ensures more accurate model evaluation while assessing a model’s generalization capability. The five-fold cross-validation method was used for this experiment. Table 4 represents the results of training accuracy, validation accuracy, and testing accuracy. The table also shows the training time that was consumed for each fold. This method achieved 99.02% average weighted training accuracy, 99.22% average weighted validation accuracy, and 98.96% average weighted test accuracy.

**Figure 5:**
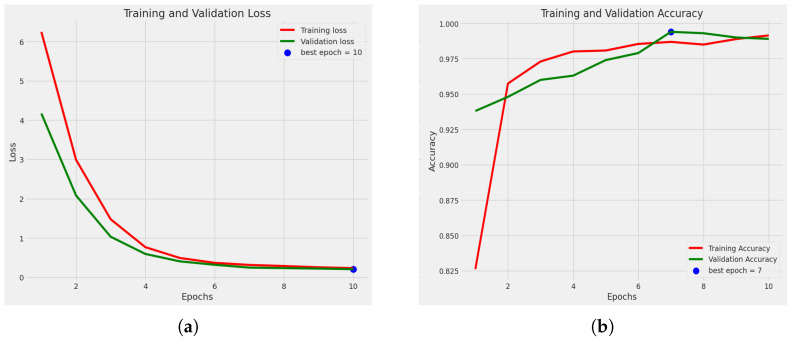
Graph of fold 3: (**a**) loss curve, (**b**) accuracy curve.

Figure 5a shows the loss curve of the best fold, which is fold 3. Figure 5b shows the accuracy curve for training and validation of the best fold. Among the five folds, this fold achieved the best accuracy. Fold 3 achieved a training accuracy of 99.14%, a validation accuracy of 99.40%, and a test accuracy of 99.50%.

**Figure 6:**
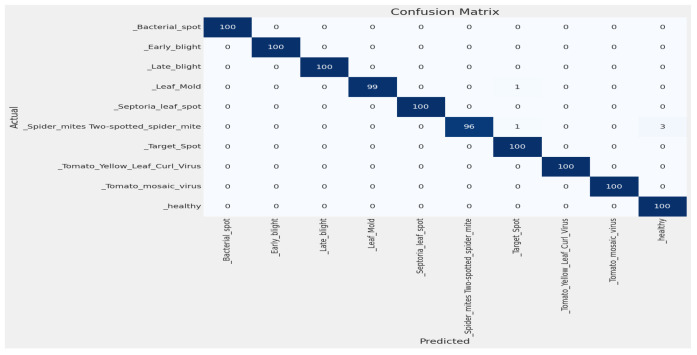
Confusion matrix of fold 3 from the five-fold cross-validation method.

Figure 6 shows the Confusion matrix of the best fold.

### 4.2. Split Method Outcome of the EfficientNetV2B2 Model

Here, all the split-method-related outcomes of EfficientNetV2B2 are fully explained.

#### 4.2.1. Statistical Analysis

**Figure 7:**
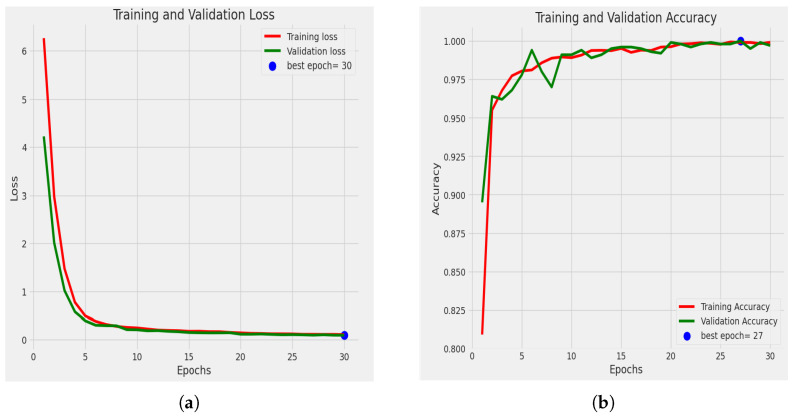
Graph of (**a**) loss curve, (**b**) accuracy graph.

Figure 7a shows the loss curve. Loss has decreased among the epochs, and the best epoch is 30 because, at epoch 30, we obtained the minimum loss, where the validation loss was 0.0942. The accuracy graph for training and validation is also shown in Figure 7b. It shows the accuracy-comparison diagram among different epochs. It shows that, as we increase the number of epochs, the accuracy percentage is also increased. At epoch 1, we obtained an accuracy of training of 80.90%, and the accuracy of validation was 89.50%. Then, at epoch 5, our training accuracy increased to 98.00%, and our validation accuracy jumped to 97.8%. After completing ten epochs, at epoch 11, we obtained an accuracy of training of 99.10% and an accuracy of validation of 99.40%. Then at epoch 26, we obtained an accuracy of training of 99.93% and an accuracy of validation of 99.80%. Then at epoch 27, the accuracy of training was 99.90%, and the accuracy of validation was 100%. And finally, at epoch 30, the accuracy of training was 99.91%, and the accuracy of validation was 99.70%. Here we obtained the greatest accuracy of training of 99.93% and accuracy of validation of 100%.

#### 4.2.2. Confusion Matrix

**Figure 8:**
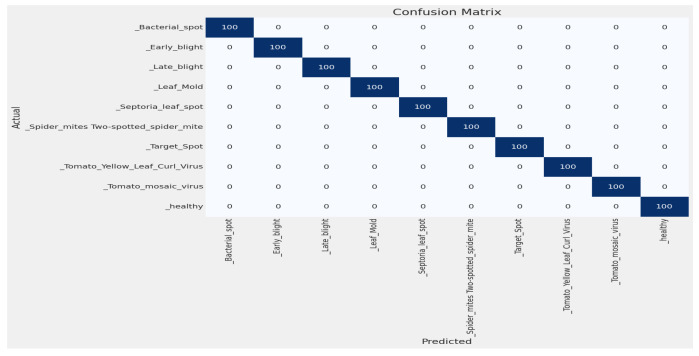
Confusion matrix for tomato leaf images.

By using this confusion matrix in Figure 8, we achieved a test accuracy of 100% for test images. As the experiment used 100 images for each class, Figure 8 explains that all the test images were predicted correctly.

#### 4.2.3. Receiver Operating Characteristic (ROC) Curve

**Figure 9:**
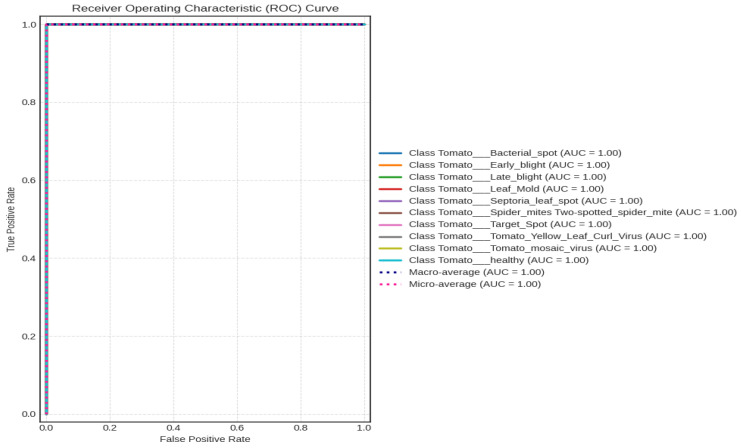
ROC curve for tomato leaf images.

A ROC curve was used to further evaluate the top-performing model, EfficientNetV2B2, as shown in Figure 9. One hundred percent is the outcome of combining the macro and micro averages.

Table 5 shows the classification report. It indicates the F1 score, recall, and accuracy. Here, the accuracy, recall, and F1 scores for all tomato image classes are 100. And also, the macro average and weighted average were 100%.

#### 4.2.4. Comparison with State-of-the-Art Models

**Figure 10:**
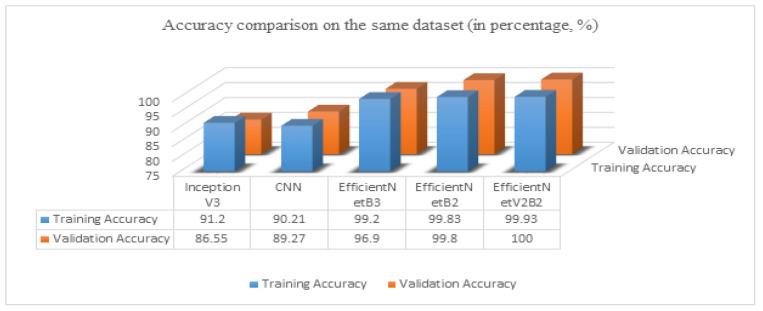
The accuracy comparison chart on the same dataset.

In Figure 10, we compare our proposed method with different models from which we experimented with our dataset [28] to know which method performs well enough. The last column represents our final approach (EfficientNetV2B2), which achieved better accuracy of training and validation than the other approaches. The proposed model EfficientNetV2B2 achieved 8.73% better training and 13.45% better validation accuracy than InceptionV3. Similarly, it achieved 9.72% better training and 10.73% better validation accuracy than CNN, 0.73% better training and 3.10% better validation accuracy than EfficientNetB3, and 0.10% better training and 0.20% better validation accuracy than the EfficientNetB2 model.

#### 4.2.5. Comparison with Customized Models on the Plant Village Dataset

**Figure 11:**
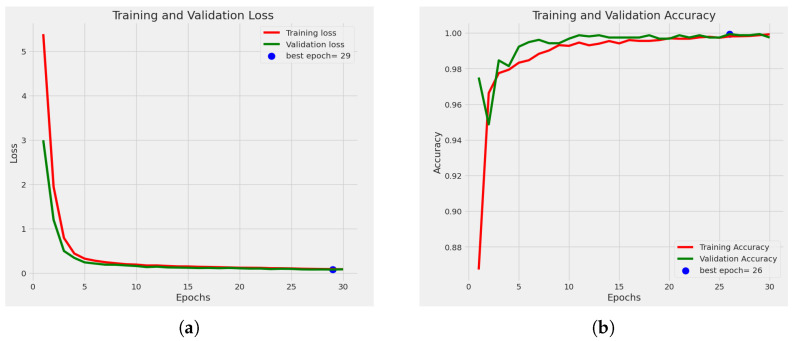
Graph of (**a**) loss curve of plant village dataset and (**b**) accuracy graph of the plant village dataset.

**Figure 12:**
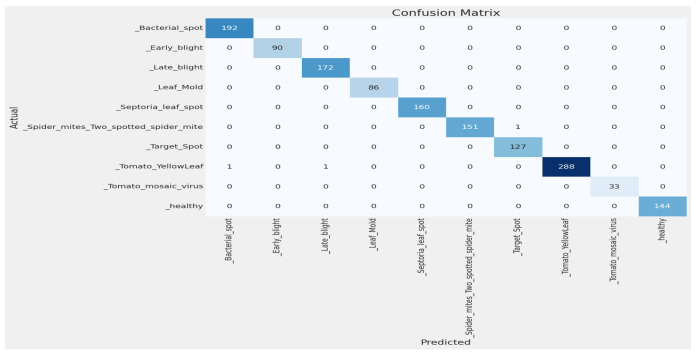
Confusion matrix of the plant village dataset.

We also tried our model with the most popular Plant Village dataset [29]. So many authors have used the dataset [29] and used only tomato leaves. So, to compare with other models, we also tried only tomato leaves from the dataset [29]. The class distribution of this dataset is shown in Table 6. The dataset is not balanced, as, in Table 6, we split the dataset [29] into (i) training (80%), (ii) validation (10%), and (iii) testing (10%). The image size used is 256 × 256. Then, we used our model EfficientNetV2B2 with an additional dense layer 256 and achieved an accuracy of training 99.92% and an accuracy of validation of 99.94%, as shown in Figure 11. Using the confusion matrix in Figure 12, we achieved a test accuracy of 99.80% for test images.

Now, we try to compare our model’s performance with other authors’ model performance on the dataset [29]. Table 7 compares our approach to the dataset [29], and we obtained the best accuracy. Table 7 shows that the proposed model achieved 2.31% better accuracy than AlexNet [33]. Similarly, it achieved 8.6% better accuracy than CNN [30], 2.99% better accuracy than SE-ResNet50 [31], 0.10% better accuracy than ResNet34 [32], 1.90% better accuracy than SECNN [34], 7.20% better accuracy than CNN [35], and 4.09% better accuracy than VGG16 [36].

### 4.3. Ablation Study

The influence of various characteristics or components on a model’s performance is examined using the research approach known as an ablation study, which is frequently employed in machine learning and scientific investigations. It entails methodically deleting or disabling particular components, such as model parameters, input characteristics, or layers, in order to gauge how each affects the system as a whole. Researchers can improve models and choose features by understanding whether parts are necessary, superfluous, or harmful through ablation experiments.

After achieving a good result in the EfficientNetV2B2 model, with an additional dense layer of 256 using only 30 epochs, we tried the same model with no extra layer. The accuracy for training was 99.71%, while for validation, it was 99.40%. And on test data, we achieved a test accuracy of 99.60%.

**Figure 13:**
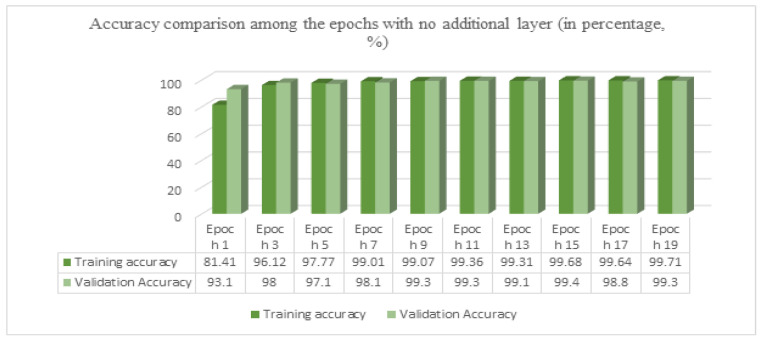
Accuracy comparison among the epochs with no additional layer.

**Figure 14:**
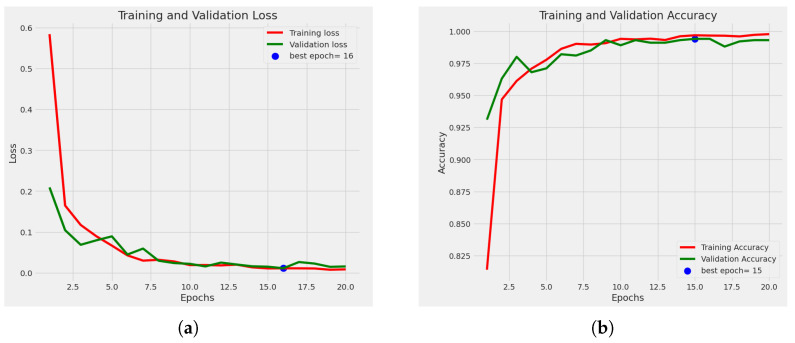
Graph of (**a**) loss curve with no additional layer, (**b**) accuracy graph with no additional layer.

**Figure 15:**
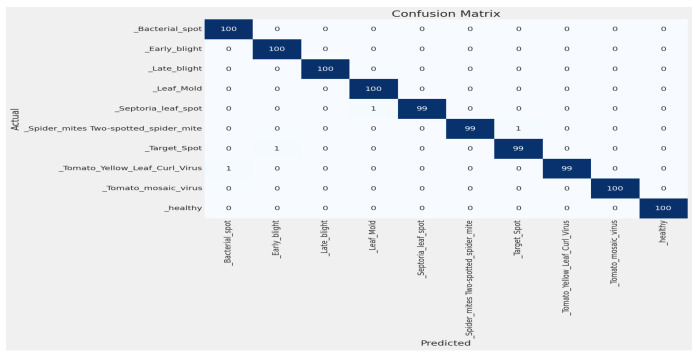
Confusion matrix with no additional layer.

In Figure 13, we used no additional layer, ran the model for 19 epochs, and stopped early at epoch 20. Figure 14a shows the loss curve, and Figure 14b shows the accuracy graph. Using the confusion matrix in Figure 15, we achieved a test accuracy of 99.60% for test images.

### 4.4. Development of Smartphone and Web Applications

**Figure 16:**
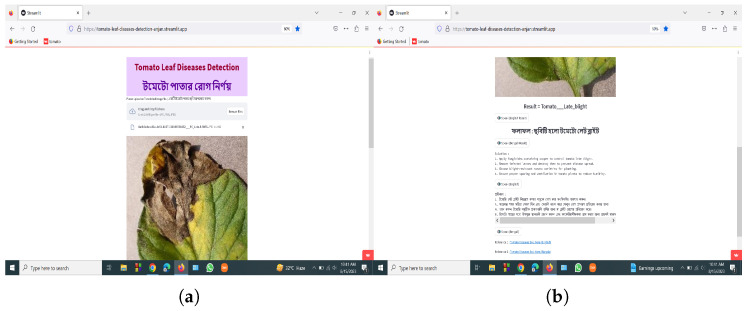
Screenshot of the web application (**a**) uploading the image, (**b**) showing the results and references.

**Figure 17:**
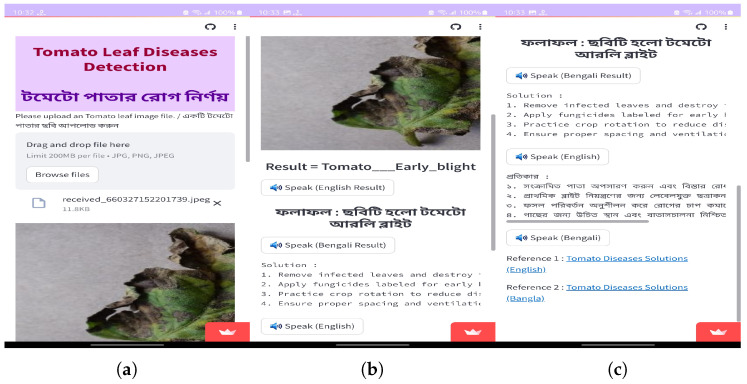
Screenshot of the smartphone application (**a**) uploading the image, (**b**) showing the results (**c**) showing rest of the results and references.

Figure 16 and Figure 17 represent the deployment result of the web application and Android application. Our web application is live on https://tomato-leaf-diseases-detection-anjan.streamlit.app/ (accessed on 2 September 2023), and our smartphone application (.apk) file is uploaded in the smartphone application folder https://zenodo.org/record/8311631 (accessed on 2 September 2023).

#### User Feedback on Applications

**Figure 18:**
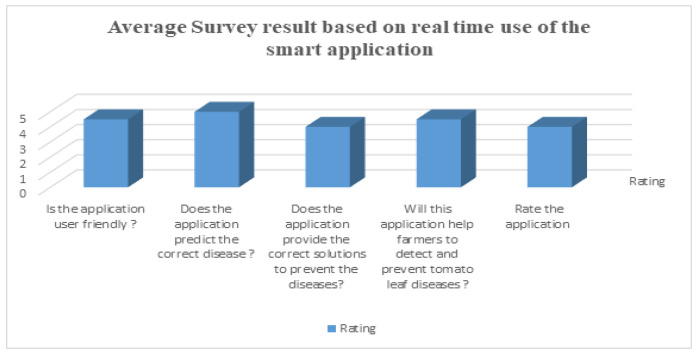
Average survey results of applications based on user feedback.

To analyze the performance of the applications, we performed this survey among some local farmers and agricultural officers who are experts in this area. They used this application for the real-time detection of tomato leaf disease and then they participated in the survey. From the survey, we obtained some average results of the survey that are shown in Figure 18. They gave ratings between 0 to 5 for each question according to their experience.

### 4.5. Discussion

This experiment was performed by using the EfficientNetV2B2 model. This experiment used the five-fold cross-validation method and the split method. The cross-validation method is very popular, and this method also performed very well on the dataset, but when the split method (80% training, 10% validation, 10% test) was used, it achieved better accuracy than the five-fold cross-validation method. We used more epochs for the five-fold cross-validation method, and it achieved better accuracy. As we used the free version of Google-colab, it has a time limitation for GPU use. The experiment with the split method achieved 100% validation accuracy and 100% test accuracy on test sets. The overfitting problem did not occur because the training set and test set are totally different. The training and validation accuracy comparison for the experiment is shown in Figure 7, and the confusion matrix is shown in Figure 8. For this experiment, we could not find any papers on our main dataset [28], so we implemented the proposed model on the very popular Plant Village dataset [29] and used only tomato leaves. We also achieved better accuracy, and a comparison is shown in Table 7. Finally, using the model, we developed smartphone and web applications to make the prediction easy.

### 4.6. Explainablity of the Proposed Model

Transparency and confidence in AI depend on its explanation. Making complicated judgments clear requires the use of deep learning models. AI and model visualization in agriculture can improve crop management, disease detection, and resource allocation. This will result in more productive and sustainable agricultural methods, which will ultimately lead to an increase in food output and global food security.

#### 4.6.1. LIME Analysis

**Figure 19:**
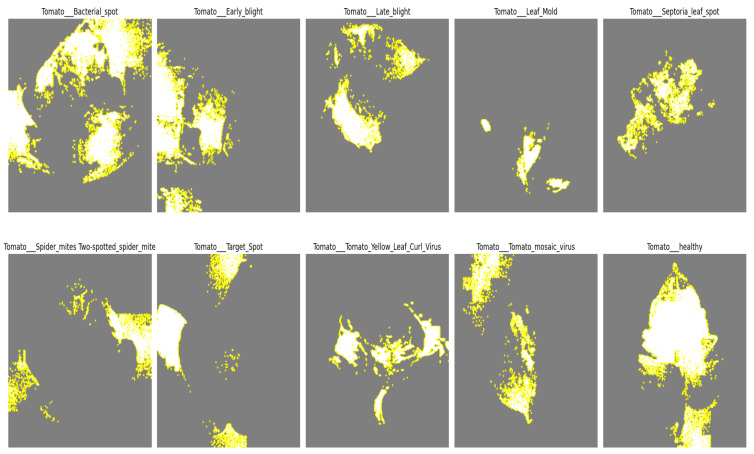
LIME experiment on each class of tomato leaves to understand the main features with the most influence on the model’s prediction.

The commonly utilized method of LIME [44] (Local Interpretable Model-agnostic Explanations) is used for understanding the predictions of complex machine learning models. LIME provides localized explanations when the model’s decision-making method is confusing or difficult to understand by essentially replicating the way the model acts around certain data examples. It accomplishes this through a novel method in which it imitates the complicated model’s behavior in close proximity to particular data instances. LIME derives important insights into why the model produced a certain prediction by perturbing and probing the input data surrounding a given sample. With the help of this comprehension tool, machine learning models may become more transparent and trustworthy, which makes it simpler for stakeholders and practitioners to comprehend and accept the model’s judgments. Figure 19 shows the output of the LIME experiment, which shows which features in an image have the most powerful influence on the model’s prediction. We can also perform error analysis and debugging using the output of LIME. Here, we can see the specific features that are influencing the model to make the correct decision about the different classes. Every class has different features to identify. It shows how our model makes decisions.

#### 4.6.2. Grad-CAM Analysis

**Figure 20:**
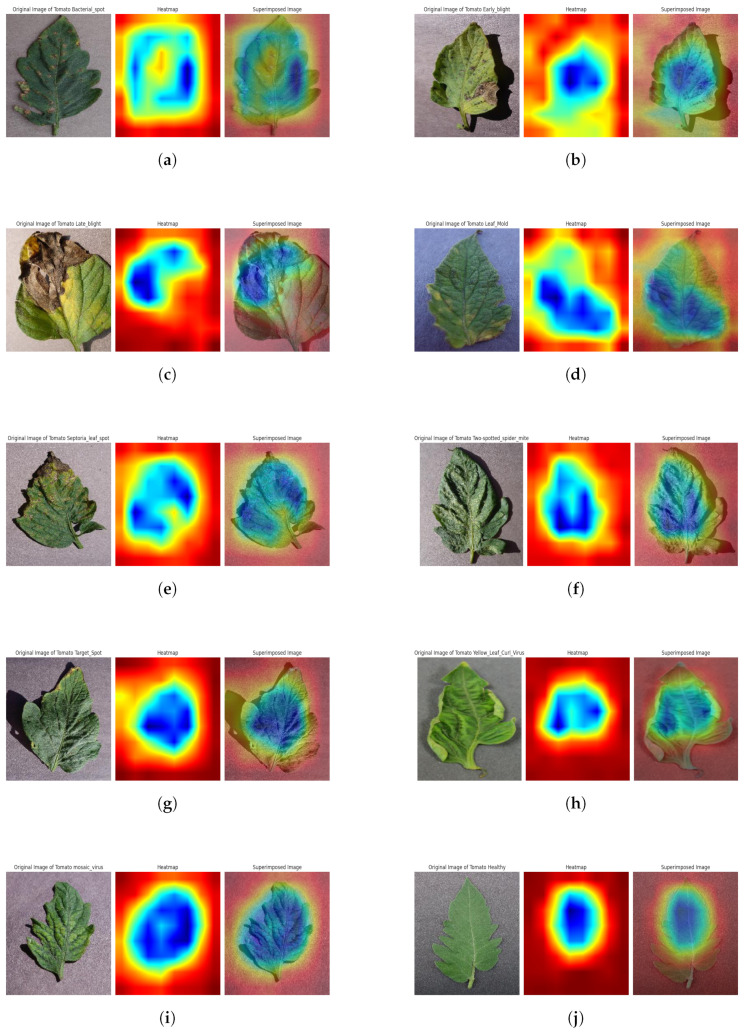
Grad-Cam visual explanations of (**a**) bacterial spot, (**b**) early blight, (**c**) late blight, (**d**) leaf mold, (**e**) septoria leaf spots, (**f**) spider mites and two-spotted spider mites, (**g**) target spot, (**h**) yellow leaf curl virus, (**i**) mosaic virus, (**j**) tomato healthy.

To give visual explanations, Grad-CAM (Gradient-weighted Class Activation Mapping) highlights the portions of the given image that are essential for the model’s decision-making. The last convolutional layer’s feature maps are used by the Grad-CAM method to take advantage of gradients in the target class score. The Grad-CAM is a useful technique for computer vision and model interpretability. Its primary use is to give users visual cues into how deep neural networks make decisions, particularly when carrying out picture classification tasks. This is accomplished by locating and emphasizing the areas of an input picture that are crucial to the model’s final categorization determination. The technique makes use of the gradients between the target class score and the feature maps produced by the last convolutional layer of the model. These gradients effectively serve as a spotlight, illuminating the areas of the picture that the model considers to be most important in making its categorization determination; this analysis’s outcome behind a certain prediction, improving the model’s interpretability, and fostering confidence in complicated neural networks are all aided by this depiction. We used the top activation layer as our target layer for the Grad-Cam visualization; this analysis’s outcome is shown in Figure 20. This helps us to verify and understand the assumptions that drive the predictions of our model. It can make the model’s decision-making process clear and understandable to both technical and non-technical uses by providing graphic explanations.

## 5. Conclusions

This experiment has presented a technique for classifying and identifying tomato leaf disease using the model EfficientNetV2B2. It contrasts the suggested method with a number of artisanal shallow structure approaches based on deep learning and machine learning. Using the dataset [28], the suggested approach produces outcomes that are encouraging in terms of accuracy. A weighted training accuracy of 99.02 percent, a weighted validation accuracy of 99.22 percent, and a weighted test accuracy of 98.96 percent were all attained using the five-fold cross-validation method. With a split-method accuracy of training of 99.93%, an accuracy of validation of 100%, and test accuracy of 100%, we demonstrated encouraging results in the experimental section. We developed a smartphone application and web application that takes an image of a tomato leaf as input and provides the correct result and solution for that disease. It supports both Bangla and English languages, a solution that will benefit farmers. There are speaking buttons that read the solutions and results for the users. This experiment has some limitations. The experiment was performed on only nine classes of diseases, and one class comprised healthy tomato leaves. The number of diseases could increase in the future. Also, this experiment only used two languages. It could be increased further. As we explained earlier, the user’s test image was stored on a server, and used the images as a training dataset, but it was a manual process for us. Our future goal is to decrease these limitations and also use this approach on other crops and analyze their performance. The study demonstrates how deep learning algorithms may promote the sustainable growth of tomato crops by revolutionizing disease-management methods. Our web and smartphone tools may be used to make this identification process simpler, more accurate, and more optimistic. This technology can help farmers make educated decisions and allocate resources efficiently, and tomato leaf disorders impact crop quality and yield with more research and integration into agricultural systems.

## Figures and Tables

**Table 1:** Summary of the literature review.

Authors	Datasets	Model	Accuracy
Agarwal et al. [30]	Plant Village [29]	Convolution Neural Network	91.20%
Ahmad et al. [37]	Laboratory-Based	InceptionV3	99.60%
Zhao et al. [31]	Plant Village [29]	SE-ResNet50	96.81%
Zhou et al. [38]	Tomato leaf disease	RRDN	95%
Trivedi et al. [39]	Tomato leaf disease	Convolution Neural Network	98.58%
Wu et al. [40]	Plant Village [29]	GoogLeNet	94.33%
Chen et al. [19]	Hunan Vegetable Institute	B-ARNet	88.43%

**Table 2:** Classes with the number of samples.

Class Names	Training Images	Validation Images	Test Images	Total Images
Mosaic Virus	800	100	100	1000
Target Spot	800	100	100	1000
Bacterial Spot	800	100	100	1000
Yellow Leaf Curl Virus	800	100	100	1000
Late Blight	800	100	100	1000
Leaf Mold	800	100	100	1000
Early Blight	800	100	100	1000
Spider Mites Two-Spotted Spider Mite	800	100	100	1000
Septoria Leaf Spot	800	100	100	1000
Healthy	800	100	100	1000

**Table 3:** Hyperparameters of EfficientNetV2B2.

Hyperparameters	Short Description
Batch Normalization	Technique used in deep learning to stabilize and accelerate training by normalizing the inputs of each layer in a mini-batch.
Learning Rate	Controls how quickly a machine learning model adapts its parameters during training.
Kernel Regularizer	Discourages excessive weight values in neural networks to prevent overfitting.
Activity Regularizer	Penalizes neural activation values to prevent overfitting in deep learning models.
Bias Regularizer	Discourages large bias values in neural networks to improve generalization and prevent overfitting.
Activation	Introduces non-linearity to model data by transforming neuron outputs.
Adamax	An optimization algorithm for deep learning, a variant of Adam.

**Table 4:** Outcome of five-fold cross-validation technique.

Fold Numbers	Training Accuracy	Validation Accuracy	Test Accuracy	Required Time (Minutes)
1	99.03%	99.20%	99.20%	36.0
2	99.01%	99.40%	98.90%	40.18
3	99.14%	99.40%	99.50%	42.56
4	98.91%	99.40%	98.10	37.10
5	99.02%	98.70%	99.10%	42.53
**Average Weighted Accuracy**	99.02%	99.22%	98.96%	39.67

**Table 5:** Performance matrix.

Name	Precision (%)	Recall (%)	F1-Score (%)	Accuracy (%)
Bacterial spot	100	100	100	100
Early blight	100	100	100	100
Late blight	100	100	100	100
Leaf Mold	100	100	100	100
Septoria Leaf Spot	100	100	100	100
Spider Mites Two-Spotted Spider Mite	100	100	100	100
Target spot	100	100	100	100
Yellow Leaf Curl Virus	100	100	100	100
Mosaic virus	100	100	100	100
Healthy	100	100	100	100
Macro average	100	100	100	100
Weighted average	100	100	100	100

**Table 6:** Distribution of classes of the plant village dataset.

Class Names	Training Images	Validation Images	Test Images	Total Images
Mosaic Virus	303	37	33	373
Target Spot	1120	157	127	1404
Bacterial Spot	1720	215	192	2127
Yellow Leaf Curl Virus	4758	310	289	5357
Late Blight	1580	157	172	1909
Leaf Mold	761	105	86	952
Early Blight	800	110	90	1000
Spider Mites Two-Spotted Spider Mite	1375	150	151	1676
Septoria Leaf Spot	1433	178	160	1771
Healthy	1287	160	144	1591

**Table 7:** Comparing our method to other methods on the plant village dataset.

Authors	Datasets	Model	Accuracy Rate	Year
Rangarajan et al. [33]	Plant Village Dataset [29]	AlexNet	97.49%	2018
Agarwal et al. [30]	Plant Village Dataset [29]	Convolution Neural Network	91.20%	2020
Zhao et al. [31]	Plant Village Dataset [29]	SE-ResNet50	96.81%	2021
Tan et al. [32]	Plant Village Dataset [29]	ResNet34	99.70%	2021
Naik et al. [34]	Plant Village Dataset [29]	SECNN	97.90%	2022
Kurmi et al. [35]	Plant Village Dataset [29]	CNN	92.60%	2022
Paymode et al. [36]	Plant Village Dataset [29]	VGG16	95.71%	2022
Proposed approach	Plant Village Dataset [29]	EfficientNetV2B2	99.80%	-

## Data Availability

The main dataset used for this experiment was collected from Kaggle which is available at https://www.kaggle.com/datasets/kaustubhb999/tomatoleaf (accessed on 30 June 2023). This research also used the plant village dataset for comparison and collected only tomato leaves. The dataset was collected from https://data.mendeley.com/datasets/tywbtsjrjv/1 (accessed on 30 June 2023). The main dataset and smartphone application (.apk file) of this study are available at https://zenodo.org/record/8311631 (accessed on 2 September 2023).

## Abbreviations

The following abbreviations are used in this manuscript:

CNN	Convolutional Neural Network
Grad-CAM	Gradient-weighted Class Activation Mapping
LIME	Local Interpretable Model-Agnostic Explanations
.H5	Hierarchical Data Format 5
DL	Deep Learning
ML	Machine Learning
TF	Transfer Learning
RGB	Red, green and blue
